# Implications of Edentulism on Quality of Life among Elderly

**DOI:** 10.3390/ijerph9010100

**Published:** 2012-01-04

**Authors:** Suely Maria Rodrigues, Ana Cristina Oliveira, Andréa Maria Duarte Vargas, Allyson Nogueira Moreira, Efigênia Ferreira e Ferreira

**Affiliations:** 1 Department of Community and Preventive Dentistry, Universidade Federal de Minas Gerais, Av. Antônio Carlos, 6627, Zip Code 31270.901, Belo Horizonte, MG, Brazil; Email: badi@univale.br (S.M.R.); vargasnt@task.com.br (A.M.D.V.); efigeniaf@gmail.com (E.F.F.); 2 Department of Operative Dentistry, Universidade Federal de Minas Gerais, Av. Antônio Carlos, 6627, Zip Code 31270.901, Belo Horizonte, MG, Brazil; Email: dangelogatil@terra.com.br

**Keywords:** aged, health of the elderly, oral health, quality of life, dentistry, dental care for aged

## Abstract

This study aimed was to test the association between quality of life and edentulism among elderly individuals in a city in southeastern Brazil. This cross-sectional study was carried out with 163 individuals aged 60 years or older, functionally independent and non-institutionalized. Data were collected with a questionnaire and oral examination. The edentulism was the dependent variable. The independent variables were sex, age, household income and quality of life (WHOQOL-Old) and their scores. To assess the association between the dependent variable and independent variables was used bivariate analysis (*p* < 0.10). Poisson regression model was performed, adjusting for age and sex. The average age of participants was 69 years (± 6.1), 68.7% were female and 52.8% were diagnosed as completely edentulous (90% CI: 0.33–1.24). When the independent variables were associated to the prevalence of edentulism, statistically significant associations were found for age (*p* = 0.03) and social participation dimension of the WHOQOL-Old (*p* = 0.08). In the Poisson regression, social participation remained statistically associated to edentulism {RP = 2.12 [90% CI (1.10–4.00)]}. The social participation proved to have a significant association to edentulism, thereby attesting to the negative effect of this condition on social aspects.

## 1. Introduction

The ageing of the population in recent decades is a common phenomenon in both developed and developing countries. This demographic transition is occurring as a result of changes in health indicators, such as a reduction in birth and mortalities rates and a longer life expectancy [1]. It is estimated that there will be approximately 34 million elderly individuals in Brazil by the year 2025, which will be the sixth largest population of elderly individuals in the World. Among the current population of approximately 170 million inhabitants, the elderly population in Brazil has surpassed 15 million individuals, corresponding to approximately 8% of the total population (6% between 60 and 74 years and 2% aged 75 years or older) [2].

The elderly population in Brazil faces considerable inequality. A large portion of these individuals have low buying power, a low degree of schooling, difficulties in access to cultural assets and healthcare and have experienced a loss or inversion of social roles. Thus, this population is more exposed to factors that compromise quality of life [3]. The investigation into aspects that allow a satisfactory quality of life among elderly individuals is of scientific and social importance. Studies of this type seek to establish associations between wellbeing and ageing, thereby contributing toward the understanding of ageing and the limits of human development [4]. 

The study of the elderly population has led to new understandings regarding the concept of quality of life, including physical/psychological/social wellbeing and self-esteem, which can be negatively affected when health is compromised. It is believed that compromised oral health can affect nutritional status, physical and mental wellbeing, pleasure in participating in an active social life and, consequently, quality of life [5].

In 1999, the World Health Organization (WHO) drafted the World Health Organization Quality of Life-Old (WHOQOL-Old) project specifically to measure quality of life in the elderly population. The aim of this project was to draft and test a generic quality of life measure for international/cross-cultural use. This tool allows assessing the impact of social and healthcare services on the quality of life of elderly individuals as well as a clearer understanding of areas of investment for achieving better gains in quality of life [6]. The aim of the present study was to test the association between quality of life and edentulism among elderly individuals in a medium-size city in Brazil.

## 2. Methods

### 2.1. Study Design and Population

A cross-sectional study was conducted in a medium-size city in southeastern Brazil. The sample was made up of male and female individuals aged 60 years or older, functionally independent, non-institutionalized, clientele of the public healthcare system. The city has 261,261 inhabitants, 21,428 of whom are elderly, representing 8.3% of the total population [7]. The city is divided into 19 urban administrative districts and has 48 Basic Healthcare Units (BHUs), 35 of which participate in the Family Health Program. A BHU is a public health clinic that provides ambulatory care for the population. Twenty-two BHUs offer ambulatory dental care carried out by oral health teams. Only two districts do not have BHUs and residents in these districts are sent to the closest healthcare unit. The total number of elderly individuals registered at the BHUs is 13,659, which corresponds to 63.7% of the total number of elderly individuals in the city.

The calculation of the sample size was based on proportion estimates, considering a 95% confidence level, 5% accuracy and 90% expected standard as well as the *M* component of the Decayed, Missing and Filled Teeth (DMFT) index on the last local epidemiological survey [8,9]. The mean of index was 24.1 (±6.0). The results of the calculation after the final correction for *n* based on the total number of elderly individuals registered in the local public healthcare system indicated a minimal sample of 163 individuals, including the 10% added to compensate for possible losses.

In order to ensure the participation of individuals from all geographical districts of the city, a proportionality calculation was performed using the total number of elderly individuals registered at each BHU. Only individuals with adequate systemic and physical conditions and the capacity to answer the questionnaire were included in the study. These conditions were assessed by the occurrence of systemic diseases, which were identified from medical charts in the archives of the BHUs and with the assistance of the BHU staff members (physician, nurse, health agent). The participants were selected randomly by lots among the elderly individuals present in the BHUs each day. 

### 2.2. Data Collection

The socio demographic data considered in the present study were sex, age and family household income. Household income was measured in terms of the Brazilian minimum wage, a standard for this type of assessment, which nearly corresponded to 315 US dollars during the period of data collection. The WHOQOL-Old questionnaire was used to measure quality of life, which was validated for use in Brazil by Fleck *et al.* (2006) [10]. The questionnaire has 24 questions grouped into six dimensions (Table 1). Each question has five Likert response options (1 to 5 points). The total score ranges from 24 to 120 points, for which higher scores denote a better quality of life. 

**Table 1:** Description of dimensions on the WHOQOL-Old.

Dimensions	Content	Questions
Functioning of senses	Impact of loss of functioning of senses on quality of life	01, 02, 10, 20
Autonomy	Independence; capability of being free to live with autonomy and make one’s own decisions	12, 13, 15, 19
Past, present and future activities	Satisfaction with life achievements and goals to be reached	03, 04, 05, 11
Social participation	Participation in activities of daily living, especially in the community	14, 16, 17, 18
Death and dying	Worries and fear regarding death and dying	06, 07, 08, 09
Intimacy	Being capable of intimate and personal relationships	21, 22, 23, 24

Source: The WHOQOL-Old Group (2005) [6].

The WHOQOL-Old was administered in interview form, considering the characteristics that are common to elderly individuals, such as difficulties in reading (visual impairment and illiteracy), understanding and marking the response. The oral examination was conducted by the researcher SMR, with the help of a trained scorer. The tests were conducted under natural light, using a wooden spatula [11]. A pilot study was carried out with eight elderly individuals receiving dental care at the teaching institution. The aim of the pilot study was to determine the adequacy of the work method, the reaction of participants, the way the questions were addressed and time spent. The participants in this phase were not involved in the main study. The study received approval from the ethics committee of the Universidade Federal de Minas Gerais (COEP-UFMG 446/07). All participants signed terms of informed consent.

### 2.3. Data Analysis

Data analysis was performed using Statistical Package for Social Sciences (SPSS for Windows, version 16.0, SPSS Inc, Chicago, IL, USA). The frequency distribution was calculated first, followed by the chi-square test (*p* < 0.10) to determine associations between the dependent and independent variables (bivariate analysis). Poisson regression analysis was employed to determine the impact of each independent variable. The independent variables were included into the multivariable model in decreasing order based on their statistical significance (*p* < 0.25/*backward stepwise procedure*) or for questions of clinical-epidemiological importance [12]. The model was adjusted for age, sex and household income. Edentulism (presence/absence) was the dependent variable. The independent variables were sex, age, household income, total WHOQOL-Old score and score on each dimension of the questionnaire.

## 3. Results

One hundred sixty-three elderly individuals participated in the study. All elderly invited to the study agreed to participate. Age ranged from 60 to 87 years, with a mean age of 69 years (±6.1). The majority of participants were women (68.7%). Eighty-six individuals were edentulous (52.8%). Just 21 elderly individuals (12.9%) reported household income of 2.0 to 6.2 times the Brazilian minimum wage per month.

Table 2 displays the distribution of scores on the dimensions of the WHOQOL-Old questionnaire associated to the presence or absence of edentulism. There were similar scores between groups, indicating that edentulism did not affect quality of life. The mean total WHOQOL-Old score was 81.0 [67.0% of maximal score on the index (120 points)], ranging 62 (51.0% of maximal score) to 98 (81.0% of maximal score). Modal and mean values confirmed a quality of life index of around 68.0% of the maximal score, with no association to edentulism.

When the independent variables were associated to the prevalence of edentulism, statistically significant associations were found for age (*p* = 0.03) and social participation (*p* = 0.08) (Table 3). Data analysis showed no statistically significant association between age and social participation (*p* = 0.74).

**Table 2:** Distribution of scores on the WHOQOL-Old dimensions according to presence or absence of edentulism among elderly individuals (n = 163).

WHOQOl-old dimension	Mean	Min.	25 percentile	Median	75 percentile	Max.	Modal
**Functioning of senses**							
Edentulous	12.1 ± 3.0	6.0	9.0	12.0	15.0	19.0	9.0
Non-edentulous	11.9 ± 3.1	7.0	9.0	12.0	15.0	20.0	16.0
**Autonomy**							
Edentulous	14.1 ± 1.6	9.0	13.0	14.0	15.0	17.0	15.0
Non-edentulous	14.1 ± 1.6	9.0	13.0	14.0	16.0	17.0	14.0
**Present/past/future activities**							
Edentulous	12.9 ± 1.8	9.0	12.0	13.0	14.0	18.0	14.0
Non-edentulous	13.1 ± 1.8	8.0	12.0	13.0	15.0	18.0	13.0
**Social Participation**							
Edentulous	14.8 ± 1.6	10.0	14.0	15.0	16.0	19.0	16.0
Non-edentulous	14.3 ± 1.8	8.0	13.0	15.0	16.0	19.0	15.0
**Death and dying**							
Edentulous	14.9 ± 3.7	8.0	12.0	16.0	17.7	20.0	16.0
Non-edentulous	14.8 ± 3.8	8.0	12.0	16.0	17.0	20.0	16.0
**Intimacy**							
Edentulous	12.2 ± 2.5	8.0	12.0	12.0	12.0	16.0	12.0
Non-edentulous	12.0 ± 2.4	7.0	12.0	12.0	12.0	16.0	12.0
**Total score**							
Edentulous	81.0 ± 8.1	62.0	75.0	81.5	88.2	98.0	89.0
Non-edentulous	80.4 ± 7.6	62.0	74.0	81.0	84.5	98.0	83.0

**Table 3:** Distribution of independent variations in relation to prevalence of edentulism among elderly individuals (n = 163).

Independent variables	Edentulism				
	Presence (%)	Absent (%)	Total (100%)	*p*-value *	Unadjusted prevalence ratio [CI]
**Sex**					
Male	23 (45.1)	28 (54.9)	51	0.18	0.63 [0.33–1.24]
Female	63 (56.3)	49 (43.8)	112		
**Age (years)**					
60–68	38 (44.7)	47 (55.3)	85	**0.03**	0.50 [0.27–0.94]
69–87	48 (61.5)	30 (38.5)	78		
**Household income**					
<2BMW	75 (52.8)	67 (47.2)	142	0.97	1.01 [0.65–1.56]
≥2BMW	11 (52.4)	10 (47.6)	21		
**Functioning senses**					
6–12	46 (50.5)	45 (49.5)	91	0.52	0.81 [0.44–1.52]
13–20	40 (55.6)	32 (44.4)	72		
**Autonomy**					
9–14	47 (50.0)	47 (50)	94	0.41	0.76 [0.41–1.43]
15–17	39 (56.5)	30 (43.5)	69		
**Present/past/future activities**					
8–13	45 (52.3)	41 (47.7)	86	0.90	0.96 [0.52–1.78]
14–18	41 (53.2)	36 (46.8)	77		
**Social Participation**					
8–15	37 (61.7)	23 (38.3)	60	**0.08**	1.78 [0.93–3.44]
16–19	49 (47.6)	54 (52.4)	103		
**Death and dying**					
8–16	64 (52.9)	57 (47.1)	121	0.95	1.02 [0.50–2.06]
17–20	22 (52.4)	20 (47.6)	42		
**Intimacy**					
7–12	67 (52.8)	60 (47.2)	127	0.99	0.99 [0.47–2.09]
13–16	19 (52.8)	17 (47.2)	36		
**Total score**					
62–81	43 (52.4)	39 (47.6)	82	0.93	0.97 [0.52–1.80]
82–98	43 (53.1)	38 (46.9)	81		

* 10% level of significance; ** BMW: Brazilian minimum wage.

Table 4 displays the results of the Poisson regression. Unlike the other variables analyzed in the model, social participation remained statistically associated to edentulism. Individuals with social participation values between 8 and 15 had a twofold greater chance of pertaining to the edentulous group {RP = 2.12 [90% CI (1.10–4.00)]}.

**Table 4:** Poisson regression model explaining the presence of edentulism in elderly individuals.

Backward stepwise model **		
Variable	Ratio	Adjusted PR* [CI]
**Social participation**	8–15	2.12 [1.10–4.00]
	16–19	1

* PR: prevalence ratio/10% level of significance; ** Adjusted for age, sex and household income.

## 4. Discussion

Edentulism was encountered in 52.8% of the participants. This finding may stem from socioeconomic factors, little or no information on oral healthcare, access and type of dental services used throughout life. Mutilating dental treatment (extraction) indicates that oral healthcare measures were either inexistent or failed wholly, reflecting decades of dentistry centered on non-conservative curative procedures. Brazilian studies demonstrate that the epidemiological situation of the elderly population in relation to edentulism is a serious problem [13,14,15,16]. The elderly form a group with nearly all their teeth extracted. This problem tends to worsen when no healthcare measures are taken aimed at offering dental care to the entire population, especially adults. Knowledge on the influence of edentulism over the quality of life of elderly individuals is important and should be produced and shared by the entire healthcare team, as good health does not exist without good oral health. All individuals should have adequate oral health that allows them to speak, chew, recognize flavors, smile, live without pain or discomfort and interrelate with others without embarrassment [17,18]. 

Although the present study may be considered consistent, it has some limitations that should be addressed. The weakness of cross-sectional studies resides in the difficulty establishing causal relations based on a cross-section in time. Moreover, it is likely that the sample may have excluded important data from individuals not included in the study, despite the randomization. Although adapted to the Portuguese language and validated in Brazil, the WHOQOL-Old questionnaire has a number of questions that are difficult to understand, which poses an obstacle to choosing the most adequate response regarding what one has experienced or felt within a specific time period. This was observed during the interviews with the elderly individuals, who often asked for the question to be repeated. 

The presence and absence of edentulism were quite homogeneous with regard to the dimensions of the WHOQOL-Old questionnaire. These data demonstrate that the tooth loss was seen as a consequence of ageing among the individuals surveyed and appears to have little or no impact on their quality of life. However, although it was not the objective of the present study, one point should be stressed: the participants only achieved 67.0% of the total possible score on the WHOQOL-Old questionnaire, which implies a 33.0% loss in quality of life.

The investigation into the quality of life of elderly individuals has received growing interest in the healthcare field. With the increase in this population, there is also an increase in the need to understand the life context of these individuals in order to provide them with a better health status through interventions capable of having a positive impact on the process of healthy ageing.

Due to its characteristic of decentralized care, the BHU is the first contact of the population with the public healthcare system and the present study was conducted in BHUs due to the frequent presence of elderly individuals in these locations. The BHU is a public place where the population receives ambulatory care. The functioning of these health units allows the continuous follow up of patients by multidisciplinary healthcare teams, with an integrated vision of the individual and emphasis on disease prevention. 

The greater participation of women during the data collection is likely related to greater care women take regarding health problems and consequent greater use of medical/dental services. According to Trentini *et al.* [19], despite have a greater need for care, men demonstrate greater acceptance of the ageing process and related health problems. Women demonstrate greater flexibility and dynamism with regard to ageing, always seeking possible remedies for daily health demands. The greater number of women may also be associated to the “feminization of ageing” phenomenon, as women account for the majority of the elderly population worldwide and have a greater life expectancy; on average, women live eight years longer than men [2]. 

The bivariate analysis revealed a significant association between edentulism and age. This result was expected, considering the progressiveness of dental caries, which account for most cases of tooth loss. Indeed, the Brazilian National Oral Health Survey reports a progressive DMFT index in the Brazilian population, considering the ages of 12 years (2.7 ± 3.12), 15 years (6.17 ± 4.82), 35 to 44 years (20.13 ± 7.74) and 65 to 74 years (27.79 ± 6.71) [13]. The DMFT index means number of teeth decayed, missing and filled [11]. It is fundamental in both clinical practice and research for oral health-related quality of life assessment tools to be more widely used. Such tools allow important outcomes for the lives of elderly individuals to be taken into account in both biological and social terms when formulating public health policies. The use of such tools could result in changes in healthcare practices and the consolidation of new paradigms in the health-disease process, with important promotion, prevention, treatment and rehabilitation actions in the healthcare field. Rehabilitation is an alternative, but should not be considered a solution. Healthcare, therefore, should have a broader scope and not be restricted to this age group, but should be a continuous effort involving younger generations and accompanying the entire lifecycle.

Analyzing the dimensions of the WHOQOL-Old, social participation was statistically associated to the prevalence of edentulism in both the bivariate and multivariate analyses. A greater percentage of respondents with a *Social Participation* score between 8 and 15 points were diagnosed as edentulous. For this population, social participation is an important factor in the perception of a better quality of life. Inadequate oral health, such as missing teeth, may have a negative influence over social activities, leading individuals to isolate themselves from others. According to Avlund *et al.* [20], regardless of age, social needs persist throughout the entire lifecycle. The majority of elderly individuals seek to maintain and broaden their social relations though group movements, leisure activities and non-formal education in an effort to promote wellbeing. 

With the ageing process, social relations undergo changes, which can play an essential role in maintaining or even improving physical and mental health. Intensive, pleasurable social experiences provided by social participation allow the formation and maintenance of friendships, which can assist in facing crises and conflict and also have an influence over self-awareness, self-esteem, personal change, learning and satisfaction with life. The positive effects of social participation are associated to the usefulness of different types of support (emotional and functional). With social support, elderly individuals generally feel loved, are confident when dealing with health problems and have higher self-esteem [21]. 

A study carried out in Brazil involving edentulous individuals found an improvement in self-image and self-esteem following the incorporation of removable complete dentures. The reports stressed the recovery of esthetics as the gateway for returning to social activities, which indicates the suffering caused by missing teeth. The patients felt “complete” again, but still considered themselves impaired [22]. 

The performance of activities of daily living may strengthen feelings of self-worth. According to the WHO (2002) [1], autonomy “is the ability of control, deal with and make personal decisions about how one should live on a daily basis in accordance with one’s own rules and preferences”, which is fundamental to the wellbeing of elderly individuals. As individuals age, their quality of life is strongly determined by their ability to maintain autonomy and independence. An adequate functional state is essential to maintaining physical and psychological wellbeing; as such activities require the interaction of cognitive resources, emotional aspects and motor skills. Physical dependence is an obstacle to general health and oral health. Individuals with a high degree of physical and/or functional dependence have a poorer oral health status.

Social participation can have a positive effect on quality of life among elderly individuals, for it provides a social support system that contributes toward minimizing feelings of loneliness and abandonment. The activities carried out appear to be an important factor, as they reinforce feeling of self-worth while also enabling personal growth [23]. Tooth loss can affect an individual’s ability to interrelate with others and can have a considerable impact on his/her lifestyle due to problems with communication in family and social interactions, causing depression, sadness, loneliness and isolation [24,25]. 

## 5. Conclusions

The prevalence of edentulism among the elderly individuals surveyed in the present study is similar to that found in the Brazilian population. Edentulism was expected to have negative consequences regarding quality of life, but did not prove to be a determinant for this outcome in this group. The social participation dimension of the WHOQOL-Old proved to have a significant association to edentulism, thereby attesting to the negative effect of this condition on social aspects. The overall WHOQOL-Old score demonstrated a 40.0% loss in quality of life in this group, indicating the need for more in-depth studies on this issue.
